# Effect of *Cistanche deserticola* on Rumen Microbiota and Rumen Function in Grazing Sheep

**DOI:** 10.3389/fmicb.2022.840725

**Published:** 2022-03-31

**Authors:** Xiaoyun Zhang, Xulei Liu, Shenghua Chang, Cheng Zhang, Wuchen Du, Fujiang Hou

**Affiliations:** State Key Laboratory of Grassland Agro-Ecosystems, Key Laboratory of Grassland Livestock Industry Innovation, Ministry of Agriculture and Rural Affairs, College of Pastoral Agriculture Science and Technology, Lanzhou University, Lanzhou, China

**Keywords:** feed additives, rumen, microflora, rumen function, *Cistanche deserticola*

## Abstract

For a long time, veterinary drugs and chemical additives have been widely used in livestock and poultry breeding to improve production performance. However, problems such as drug residues in food are causing serious concerns. The use of functional plants and their extracts to improve production performance is becoming increasingly popular. This study aimed to evaluate the effect of *Cistanche deserticola* in sheep feed on rumen flora and to analyze the causes to provide a theoretical basis for the future use of *Cistanche deserticola* as a functional substance to improve sheep production performance. A completely randomized experimental design was adopted using 24 six-month-old sheep males divided into four groups (six animals in each group) which were fed a basic diet composed of alfalfa and tall fescue grass. The *C. deserticola* feed was provided to sheep at different levels (0, 2, 4, and 6%) as experimental treatments. On the last day (Day 75), ruminal fluid was collected through a rumen tube for evaluating changes in rumen flora. The test results showed that *Prevotella_1*, *Lactobacillus*, and *Rikenellaceae_RC9_gut_group* were the dominant species at the genus level in all samples. *Lactobacillus*, *Rikenellaceae_RC9_gut_group*, *Ruminococcaceae_NK4A214_group*, *Butyrivibrio_2*, and *Christensenellaceae_R-7_group* differed significantly in relative abundance among the treatment groups. The polysaccharides in *C. deserticola* was the major factor influencing the alteration in rumen flora abundance, and had the functions of improving rumen fermentation environment and regulating rumen flora structure, etc. Hence, *C. deserticola* can be used to regulate rumen fermentation in grazing sheep to improve production efficiency.

## Introduction

Veterinary drugs and chemical additives not only have the potential to solve the problems heat and cold resistance and low epidemic resistance of livestock and poultry, but also improve production performance and promote growth. However, their long-term usage can lead to drug residues in livestock and poultry products, which can harm human health through food enrichment. At the same time, the residues in livestock and poultry manure can also cause secondary pollution to soil and water bodies, seriously interfering with the stability of the ecological system in fragile areas (Chi et al., 2020). With improved life quality and food consumption at the global level, environmental pollution and drug residues in food production have become a matter of great concern, and the consumers are paying more attention to food quality and safety aspects. The use of traditional methods of relying on chemical drugs for improving livestock production performance is becoming increasingly less appealing (Li et al., 2021; Wemette et al., 2021). Recently, the outstanding contribution of functional plants in medicine and health have received the attention of breeders. The green, safe and residue-free functional plant substitutes for feed additives have become the mainstream choice to promote livestock production (Bodas et al., 2012; Allen et al., 2013; Elghandour et al., 2018). Therefore, prospects for studying and developing new functional plants to replace veterinary drugs and other chemicals represent an urgent matter in animal agriculture worldwide (Mendel et al., 2017).

Compared with veterinary drugs and chemical additives, functional plants have good growth promotion, bactericidal, antioxidant and other functions, and are the rich sources of active ingredients particularly, alkaloids, and sugars that can be used to a certain extent instead of antibiotics (Wang, 2021). In addition, they have several other advantages, including the wide availability of raw materials, low cost, low residual effect, no drug resistance or toxic side effects. Several studies have shown that functional plants have an impact on ruminant immunity and ruminal fermentation parameters. For instance, it has been shown that tannins in cassava can increase water and nitrogen intake of goats as well as the digestibility of crude protein and neutral detergent fiber, thereby improving feed utilization (Nascimento et al., 2021). The addition of flaxseed in the diet of lactating dairy cows increase the content of desirable fatty acids in milk (Soita et al., 2003). The use of plantain and garlic leaf promoted healthy growth and lean meat production in sheep (Redoy et al., 2020), while, Astragalus was shown to increase dry matter intake and immunity as well as promote body weight gain of Tibetan sheep (Wang et al., 2021).

*Cistanche deserticola* is a functional plant widely found in the deserts, with anti-aging, anti-oxidation (Gao et al., 2015), immune regulation, neuroprotective (Wang et al., 2012) and liver protective effects (Morikawa et al., 2010), having been artificially cultivated at large amounts. in In addition to amino acids and inorganic trace elements, it’s also contain a wide range of other chemical components, including phenylethanol glycosides, polysaccharides, flavonoids, cyclic enol ether terpenoids, lignans, oligosaccharides, galactitols, alkaloids and volatile components, etc. (Jiang and Tu, 2009; Lai et al., 2016), which were shown to regulate the diversity of intestinal microbiota, hence, promoting intestinal health (Fu et al., 2020). Recently, few researchers have studied the chemical composition and pharmacological activity of *C. deserticola* on hormone regulation, laxative, immunomodulation, antioxidant and prevention of Alzheimer’s disease, neuroprotection, improvement of microcirculation and influence on intestinal flora, etc. (Cai et al., 2010; He et al., 2020; Yang et al., 2021; Wei et al., 2022). Most of the research objects are human and mice, while less research has been conducted on ruminants. Polysaccharide is one of its main active ingredients, has the effect of inhibiting the growth of bacteria and fungi, resisting plant and animal pathogens, while promoting the growth of a variety of probiotics, and conducive to the health of intestinal flora (Yao et al., 2019; Deng et al., 2020). A recent study by our research team has shown that *C. deserticola* has the potential to improve the growth performance and digestion of grazing sheep (Liu et al., 2020). However, the rumen being an important nutrient digestion organ of ruminants, how feeding *C. deserticola* will affect its microbiota and the rumen fermentation function is still not well understood. Therefore, in this study, the effects of different levels of *C. deserticola* fed to grazing sheep was investigated on rumen microbiota and rumen function in sheep. The results of this study are expected to provide a theoretical basis for the development and use of *C. deserticola* and its by-products for ruminant use.

## Materials and Methods

### Experimental Site

This experiment was carried out at the Linze Grassland Agricultural Experiment Station of Lanzhou University, Linze County, Zhangye City, Gansu Province (100°02′ E, 39°15′ N). The dominant type of agricultural system is a specialized intensive cropping production system (SICP) and an extensively integrated crop–livestock production system (EICL) (Liu et al., 2020). The research station is located at an altitude of 1,390 m, characterized by a temperate continental desert steppe climate (Li et al., 2008). The region has a distinct dry climate with scarce rainfall. The average annual rainfall is about 121.5 mm and the annual average temperature is 7.16°C. According to the comprehensive sequential classification of grassland, the area is a mild arid temperate semi-desert meadow (Ren, 2008).

### Experimental Animals and Group Design

A completely randomized experimental design was adopted. Twenty-four 6-month-old sheep (male) with good body condition and weight (an average of 27.5 ± 5 kg) were selected and randomly divided into 4 groups with no significant difference according to their weight. Four different levels of *C. deserticola* were provided to the sheep as the treatments, including 0% (control, CON), 2% (low-level, LC), 4% (medium-level, MC) and 6% (high-level, HC). Each treatment comprised of 6 sheep. The basic diet consisted of 60% alfalfa and 40% tall fescue (DM basis) after rough cutting. which was fed in the morning (07:00), mid (12:00), and evening (19:00), and *C. deserticola* was fed once a day. Among them, the pre-feeding period was 14 days and the official period was 60 days (including 42 days for digestion test in metabolic cages and 18 days for methane production test in respiratory chambers). During the test period, all sheep were raised individually in captivity and freely existed licking salt and drinking water, natural light and cool environment temperature.

### Sample Collection and Processing

The animal care and experimental procedures were approved by the Animal Use and Care committee of Lanzhou University (Gansu, China, No. 2010-1 and 2010-2). Rumen fluid samples were taken from each sheep 2 h post-fresh forage and *C. deserticola* supply in the morning, which were collected through the oral cavity using a rumen tube, a part of the supernatant was collected by filtration with sterile 4-layer gauze, and aliquoted into a 5 ml centrifuge tube and stored at −20°C. It is used to measure the concentration of volatile fatty acids (VFA); one part was aliquoted into a 50 ml centrifuge tube for pH determination; one part was placed in a 5 ml cryotube and stored at −80°C for the determination of rumen microbiota. *C. deserticola* was purchased from a herb company, nutrient composition of *C. deserticola* and fresh forage with reference to published articles (Liu et al., 2020) by our team sample determination.

#### Extraction of DNA and 16S rDNA Sequencing

DNA was extracted from rumen samples by using the TINamp Stool DNA Kit (TIANGEN, Beijing, China) these DNA samples were used as the templates for 16S rDNA and sequencing analyses. The DNA quality and concentration was checked with Thermo NanoDrop One. For the 16S rDNA gene amplicon sequencing, the primers 515 F (5′-ACTCCTACGGGAGGCAGCA-3′) and 806R (5′- GGACTACHVGGGTWTCTAAT-3′) (targeting bacterial) – with a barcode at the 5′end of primer 515F – were used to amplify the V3-V4 region of the 16S rDNA gene. The PCR mixture contained 25 μL of 2× Premix Taq, 1 μL of Primer-F, 1 μL of Primer R, 50 ng of DNA, 50 μL of Nuclease-free water. The PCR amplification program consisted of an initial denaturation at 94°C for 5 min, followed by 30 cycles of 94°C for 30 s, 52°C for 30 s, and 72°C for 30 s, with a final extension at 72°C for 10 min. The PCR products were subjected to electrophoresis with 1%-(w/v)-agarose gel. The band was excised and purified with the E.Z.N.A.^®^ Gel Extraction Kit (Omega, United States). The sequence library was constructed with the NEB Next ^®^ Ultra ™ DNA Library Prep Kit for Illumina ^®^ (New England Biolabs, United States) and sequenced using a Illumina Nova 6000 platform (Guangdong Magi gene Biotechnology Co., Ltd., Guangzhou, China).

#### Sequencing Data Processing and Analysis

The Raw Reads data at both ends were cut by sliding window quality using FASTP (version 0.14.1) separately. Based on the primer information at the first and last ends of the sequence, the primers were removed using CUTADAPT software (version 1.14) to obtain the quality-controlled paired-end Clean Reads. Based on the overlap relationship between paired-end Clean Reads, the original splice sequences were obtained by filtering the non-conforming tags with USEARCH -FASTQ_MERGEPAIRS (version 10.0.240). Use FASTP (version 0.14.1) to perform sliding window quality clipping on Raw Tags data to obtain Clean Tags for further analysis. UPARSE in USEARCH 10 (version 10.0.240) software was used to cluster Tags at 97% similarity level to obtain OTUs. Then, we performed taxonomy annotation on OTUs, Representative sequences were classified into organisms using RDP classifier (version 11.5) based on the SILVA (version 138) database. Alpha diversity analysis (richness, chao1, shannon_2, simpson) were calculated using USEARCH -ALPHA_DIV (version 10.0.240). PCoA (Principal coordinate analysis) was performed using the vegan package of R software with a distance algorithm. LEfse and LDA Based on OTU abundance tables, Kruskal Wallis rank-sum test was performed using R software, followed by FDR correction, and then significant analysis of species differences between groups was performed using LEfse software based on the homogenized abundance tables for each species class. Based on 16S sequencing data, the rumen microbiota functional pathways were predicted using Tax4Fun based on the information from the KEGG database. The correlation heatmaps were generated using the R program heatmap package. The relative abundance of bacteria and the alpha diversity indices were analyzed using a completely randomized design by one-way analysis of variance. Significant difference was declared at *P* < 0.05.

## Results

### Impact of *Cistanche deserticola* Addition on the Diversity of Sheep Ruminal Microbiota

#### Number of Operational Taxonomic Unit

**FIGURE 1:**
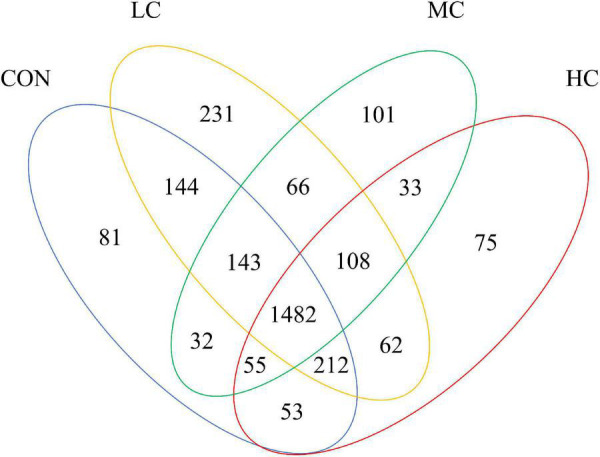
Venn diagram of ruminal fluid flora.

High-throughput sequencing was performed on 24 sheep ruminal fluid samples. Raw sequences were spliced and filtered. All samples yielded 1,186,925 raw reads, with an average of 49,455 reads for each sample (largest read: 67,681; smallest read: 35,903). Based on the identification of 97% of nucleotide sequences from total reads, identified 8,747 OTUs (Figure 1). A total of 1,482 OTUs were shared between samples from different group. Unique OTU numbers in CON, LC, MC, HC were 81 (0.93%), 231 (2.64%), 101 (1.15%), 75 (0.86%), respectively. The highest number of OTUs was found in LC, and the lowest number in HC.

#### Diversity Analysis

##### α Diversity Analysis

Alpha diversity index analysis was performed on the four treatment groups of CON, LC, MC, and HC. The results showed that the richness index and Chao1 index of the LC group were significantly higher than the CON, MC, and HC groups (Table 1), this indicates that the richness of microbial communities in samples from LC group was significantly higher than other groups, and the Shannon_2 index of CON and LC groups was significantly higher than MC and HC, while the Simpson index was significantly lower than MC and HC (Table 1), indicating that the diversity of microbial communities in CON and LC groups was significantly higher than MC and HC. In conclusion, we infer that the addition of a low level of *C. deserticola* significantly increased the richness and diversity of microbial communities in the rumen fluid of sheep.

**TABLE 1:** Alpha diversity index.

Items	CON	LC	MC	HC	SEM	*P*-value
Richness index	1167^b^	1548^a^	977^b^	1093^b^	282.665	0.001
Chao1 index	1169.2^b^	1548.8^a^	979.5^b^	1095.1^b^	282.129	0.001
Shannon_2 index	6.50^a^	7.33^a^	5.00^b^	5.38^b^	1.227	0.001
Simpson index	0.039^b^	0.025^b^	0.126^a^	0.135^a^	0.068	0.001

*CON, C. deserticola at 0%; LC, C. deserticola at 2%; MC, C. deserticola at 4%; HC, C. deserticola at 6%. ^a^ and ^b^ mean within the same row with the different letters are significantly different (P < 0.05).*

##### β Diversity Analysis

**FIGURE 2:**
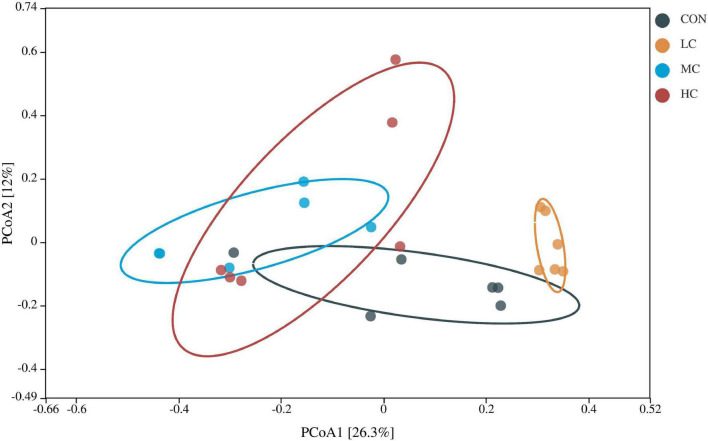
Principal coordinates analysis of rumen microbiota in different treatment groups.

β diversity analysis was used to determine the structural differences in microbial communities among samples from different treatment groups. As shown in Figure 2, the distribution of samples from CON, MC, and HC groups was discrete and cross-over and overlapping with each other, while the distribution of samples from LC group was relatively concentrated and spatially distant from the other groups, which indicated that the samples from LC group had good repeatability and ruminal microbial communities were different from the other groups.

#### Microbial Community Differences Between Control, High-Level, Medium-Level, and Low-Level

**FIGURE 3:**
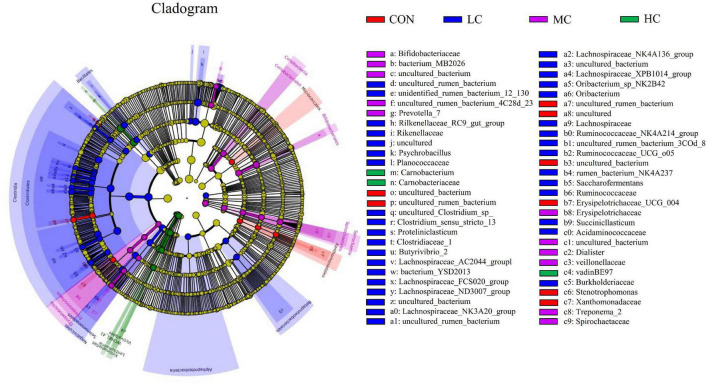
LEfSe (linear discriminant analysis Effect Size) cladogram comparing microbial communities among the three elevations. Differences are represented by the color of the group where taxa are most abundant; Red: Taxa abundant in CON, Green: Taxa abundant in HC, Purple: Taxa abundant in MC, Blue: Taxa abundant in LC. CON, *C. deserticola* at 0%; LC, *C. deserticola* at 2%; MC, *C. deserticola* at 4%; HC, *C. deserticola* at 6%.

**FIGURE 4:**
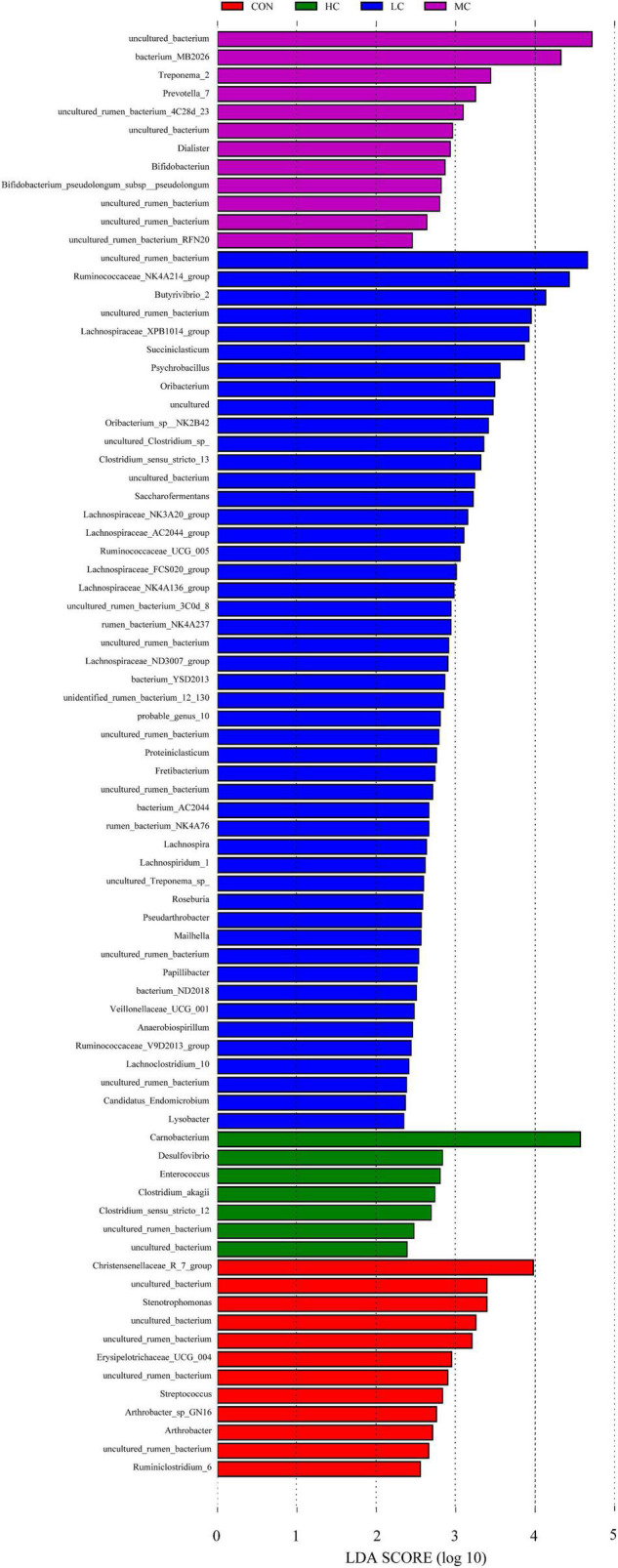
Histogram of LDA score calculated for each taxon ranging from phylum to genus. The LDA scores represent the difference in relative abundance with an exponential fold change of 10 between both communities, indicated by the significant difference in taxa. Red: Taxa abundant in CON, Green: Taxa abundant in HC, Purple: Taxa abundant in MC, Blue: Taxa abundant in LC. CON, *C. deserticola* at 0%; LC, *C. deserticola* at 2%; MC, *C. deserticola* at 4%; HC, *C. deserticola* at 6%.

The LEfSe (linear discriminant analysis Effect Size) was performed to detect variations in the bacterial taxa composition. Figure 3 depicts a representative cladogram of the structure of the predominant microbiome, showing the most remarkable differences in taxa among the different additional levels. Data indicate that eight clades were more abundant in the CON group, thirty-four clades were more abundant in the LC group, eleven clades were more abundant in the MC group, and three clades were more abundant in the HC group. The abundance differences of different bacterial groups among CON, HC, MC, and LC are shown in Figure 4. Among them, the most differential bacterial genus in CON is *Christensenellaceae_R_7_group*, and the most differential bacterial genus in HC is *Carnobacterium*. *Ruminococcaceae_NK4A214_group*, *Butyrivibrio_2*, and *Succiniclasticum* were more abundant in LC, and among them, *Carnobacterium* and *Ruminococcaceae_NK4A214_group* had the largest difference among communities, with the absolute score of LDA being ∼ 5.

#### Analysis of Bacterial Composition and Community Structure

**FIGURE 5:**
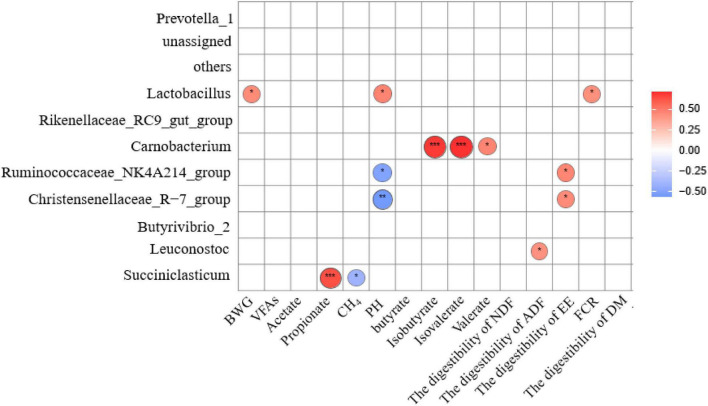
Spearman correlation and clustering analysis between Rumen fermentation parameters, rumen digestive metabolism, Growth Performance and main bacteria in Genus level. The area of the circle represents the magnitude of the correlation, and different colors represent either a positive correlation (red) or a negative correlation (blue), *represents 0.01 < *P* ≤ 0.05, **represents *P* ≤ 0.01, ***represents *P* ≤ 0.001. BWG, bodyweight gain; FCR, feed conversion ratio (the ratio of BWG divided by the total DMI); VFAs, total volatile fatty acid; EE, ether extract.

**FIGURE 6:**
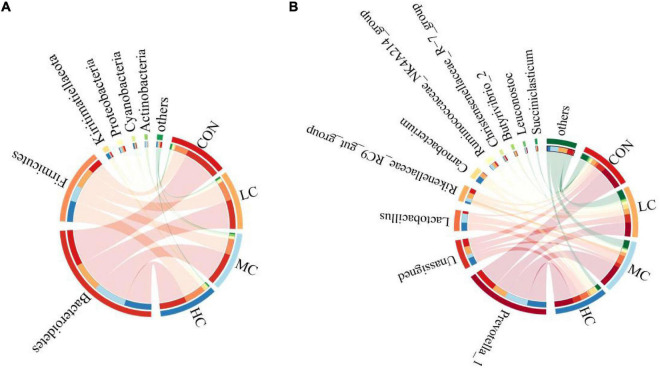
Bacterial phyla **(A)** and genus **(B)** (relative abundance >1%) of 4 treatments visualized using Circos.

The species composition and relative abundance of the sheep ruminal microbiota with the relative abundance above 1% and the first 15% both at phylum and genus were shown in Table 2. Figure 5 represents the microbiota with the significant difference in species composition among groups at the genus level and the analysis of their proportions in each group. At the phylum level, Bacteroidetes, Firmicutes, Kiritimatiellaeota, Proteobacteria, Cyanobacteria, and Actinobacteria were mainly detected. The main phyla of microorganisms in the ruminal fluid of sheep in the four treatment groups were Bacteroidetes, Firmicutes, Kiritimatiellaeota, and Proteobacteria, which accounted for 94.62% of all bacteria (Table 2 and Figure 6A). At the genus level, *Prevotella_1*, *Lactobacillus*, *Rikenellaceae_RC9_gut_group*, *Unassigned*, *Carnobacterium*, *Ruminococcaceae_NK4A214_group*, *Christensenellaceae_R-7_group*, *Butyrivibrio_2*, *Leuconostoc*, *Succiniclasticum* were mainly detected. The main microbial genera in the ruminal fluid of sheep in the four treatment groups were *Prevotella_1*, *Lactobacillus*, *Rikenellaceae_RC9_gut_group*, and *Unassigned* (Table 2 and Figure 6B).

**TABLE 2:** Species and relative abundance of ruminal fluid microbiota in sheep at phylum level and genus level.

Bacterial taxa	CON	LC	MC	HC	SEM	*P*-value		
						ANOVA	Linear	Quadratic
**Phylum level**								
Bacteroidetes	0.664^a^	0.534^ab^	0.595^ab^	0.496^b^	0.125	0.083	0.213	0.038
Firmicutes	0.244	0.367	0.290	0.383	0.123	0.185	0	0.078
Kiritimatiellaeota	0.022^b^	0.012^b^	0.011^b^	0.059^a^	0.023	0.000	0.020	0.625
Proteobacteria	0.024	0.035	0.037	0.011	0.025	0.290	0.691	0.768
Cyanobacteria	0.011^ab^	0.020^b^	0.006^ab^	0.026^a^	0.014	0.050	0.918	0.336
Actinobacteria	0.005	0.005	0.041	0.003	0.044	0.404	0.237	0.592
Others	0.030	0.029	0.021	0.022	0.017	0.764	0.206	0.462
**Genus level**								
*Prevotella_1*	0.456	0.299	0.463	0.382	0.142	0.165	0.258	0.263
*Unassigned*	0.163^a^	0.169^a^	0.079^b^	0.163^a^	0.066	0.050	0.511	0.416
*Lactobacillus*	0.050^ab^	0.004^b^	0.185^a^	0.172^a^	0.143	0.068	0	0.294
*Rikenellaceae_RC9_gut_group*	0.093^ab^	0.120^a^	0.066^ab^	0.043^b^	0.048	0.028	0.521	0.471
*Carnobacterium*	0.009	0.091	0.002	0.130	0.109	0.121	0.838	0.206
*Ruminococcaceae_NK4A214_group*	0.030^b^	0.059^a^	0.059^a^	0.009^c^	0.024	0.000	0.035	0.609
*Christensenellaceae_R-7_group*	0.025^a^	0.016^ab^	0.007^b^	0.011^ab^	0.013	0.097	0.141	0.026
*Butyrivibrio_2*	0.010^b^	0.036^a^	0.005^b^	0.005^b^	0.015	0.000	0.560	0.453
*Leuconostoc*	0.018	0.000	0.023	0.004	0.025	0.373	0.318	0.475
*Succiniclasticum*	0.015	0.020	0.003	0.006	0.018	0.378	0.824	0.515
Others	0.133^ab^	0.186^a^	0.164^ab^	0.076^b^	0.078	0.077	0.474	0.462

*CON, C. deserticola at 0%; LC, C. deserticola at 2%; MC, C. deserticola at 4%; HC, C. deserticola at 6%. ^a, b^, and ^c^ mean within the same row with the different letters are significantly different (P < 0.05).*

At the phylum level, adding *C. deserticola* had no significant effect on the relative abundance of Firmicutes, Proteobacteria, and Actinobacteria in the ruminal fluid of sheep as compared to the CON group. The relative abundance of Bacteroides in the HC group was significantly lower than that in the CON group, the relative abundance of Kiritimatiellaeota was significantly higher than that in the other groups, while the relative abundance of Cyanobacteria was significantly higher than that in the LC group (Table 2 and Figure 6A).

At the genus level, compared with the CON group, adding *C. deserticola* had no significant effect on the relative abundance of *Prevotella_1*, *Carnobacterium*, *Leuconostoc*, and *Succiniclasticum* in sheep ruminal fluid (Table 2). Compared with the group without *C. deserticola*, *C. the deserticola* at 2 and 6% significantly increased the relative abundance of *Unassigned* (Table 2). Moreover, adding *C. deserticola* at 4 and 6% significantly increased the relative abundance of *Lactobacillus* (Table 2) *C. deserticola* at 2% increased the relative abundance of *Rikenellaceae_RC9_gut_group*, *Ruminococcaceae_NK4A214_group*, and *Butyrivibrio_2* (Table 2); Adding *C. deserticola* significantly lessened the relative abundance of *Christensenellaceae_R-7_group* (Table 2).

### Rumen Fermentation Parameters, Rumen Digestive Metabolism, Growth Performance Concerning Main Bacteria in Genus Level

At the genus level, pH, BWG, and FCR were positively correlated with the relative abundance of *Lactobacillus* (*P* < 0.05). The proportion of propionic acid in the ruminal fluid was significant positive correlated with the relative abundance of *Succiniclasticum* (*P* < 0.01) while CH_4_ production was negatively correlated with the relative abundance of *Succiniclasticum* (*P* < 0.05). The pH was negatively correlated with the relative abundance of *Ruminococcaceae_NK4A214_group* (*P* < 0.05) and *Christensenellaceae_R-7_group* (*P* < 0.01). Significant and positive correlation between the proportion of Isobutyrate acid (*P* < 0.01), Isovalerate acid (*P* < 0.01), and Valerate acid (*P* < 0.05) with the relative abundance of *Carnobacterium* were detected. Also, a significant positive correlation was found between the digestibility of ADF with the relative abundance of *Leuconostoc* (*P* < 0.05), and that of EE with the relative abundance of *Ruminococcaceae_NK4A214_group* and *Christensenellaceae_R-7_group* (*P* < 0.05).

### Tax4Fun Gene Functions Estimation

**FIGURE 7:**
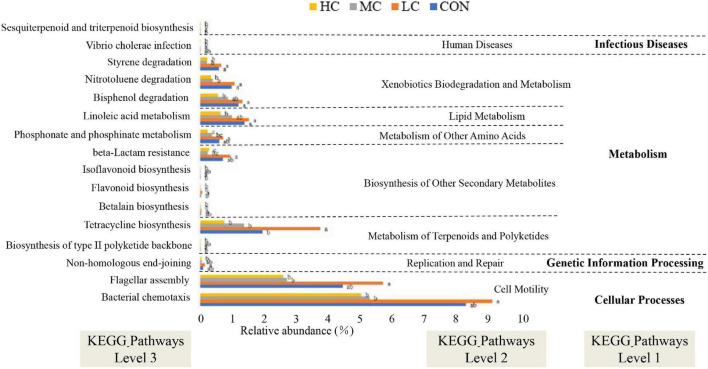
Functional predictions for rumen microbiota with significantly different KEGG pathways (*P* < 0.05) for the four add levels (CON, HC, MC, and LC). KEGG pathways at Level 1, Level 2, and Level 3 are represented. CON, *C. deserticola* at 0%; LC, *C. deserticola* at 2%; MC, *C. deserticola* at 4%; HC, *C. deserticola* at 6%. In each group, different lowercase letters show a significant difference (*P* < 0.05), while the same letters show no significant difference (*P* > 0.05), respectively.

Tax4Fun was used to predict the function of microbial communities in the rumen of sheep. Notably, we obtained 174 predictive functions, and some of the functions with significant differences are shown in Figure 7, more functions with differences were related to “metabolism” functions, among which “sesquiterpenoid and triterpenoid biosynthesis” functional genes were enriched in LC group sheep, suggesting that the synthesis of flavonoids and terpenes might be favored in the rumen of sheep.

## Discussion

The rumen is a complex microbial anaerobic fermentation chamber that harbors one of the most diverse intestinal microbial communities of the animal kingdom (Li et al., 2021). In terms of quantity and variety, bacteria are the dominant species in the rumen, and the catabolic potential of ruminal bacteria plays a vital role in the health and nutrition of their host (Klassen et al., 2021). Higher microbial diversity in the mammalian gastrointestinal system often equates with stronger metabolic capacity and stability (Li et al., 2019), such as Plateau pika (Li et al., 2019). The increase of the Rumen microorganism diversity in highland yak reflects its high ability to use high-fiber pasture (Fan et al., 2020). In this study, high-throughput sequencing and β diversity analyses showed that microbial diversity was increased in both LC and MC groups. Interestingly, the abundance and diversity of ruminal microbiota were the highest in the LC group. Based on these findings, it can be speculated that sheep in the LC group might have higher digestive and metabolic capacities as well as the highest efficiency of feed utilization.

The ruminal microbiota develops from birth, thereby affecting nutrient balance, digestion, and metabolism of ruminants. The rumen can therefore be regarded as a metabolic organ with a role in protection, immunity, development, and nutrition to ruminants (Gill et al., 2006; Methé et al., 2012). The pH of the ruminal fluid is an important ruminal fermentation parameter, and the appropriate pH maintains the stability of the ruminal environment, ensuring adequate microbial activities (Pilajun et al., 2010). Rumen microorganisms can rapidly ferment carbohydrates to produce VFA and organic acids, thus lowering ruminal pH. Previous results showed that pH increased significantly with the increasing percentage of *C. deserticola* addition (Liu et al., 2020). Combined with the changes in rumen flora abundance and diversity in the present study, we infer that some components of *C. deserticola* altered rumen fermentation parameters in sheep, which in turn affected rumen flora structure.

Polysaccharides and phenethyl alcohol glycosides are the main active components of *C. deserticola* (Jiang and Tu, 2009). It was found that phenethyl alcohol glycosides play an active role mainly in immunity (Luo et al., 2020). The functional polysaccharides such as Hericium erinaceus polysaccharides, Ganoderma lucidum bran polysaccharide has positive effects in improving the microbial fermentation and rumen fermentation environment in goats (Wu, 2014; Meng, 2018; Li et al., 2019). Correlation analysis showed that *Lactobacillus* had a positive effect on rumen fluid pH, and the relative abundance of *Lactobacillus* increased significantly with the increase in proportion of *C. deserticola* addition. The growth and multiplication of beneficial bacteria such as *Lactobacillus* and *Enterococcus* in the gastrointestinal tract can inhibit the growth of harmful aerobic bacteria, thereby regulating the balance of the gastrointestinal microbiota, facilitating nutrient absorption, and improving feed utilization (Xie et al., 2020). Also, it has been found that the use of *Lactobacillus* as probiotics in ruminant feed can effectively reduce the abundance of pathogenic bacterial species in the rumen, such as *Escherichia coli* (Galvao et al., 2005), thereby increasing dry matter intake (Arowolo and He, 2018), reducing the frequency of gastrointestinal disorders (such as diarrhea) (Signorini et al., 2012), as well as by secreting bacteriocins and strengthening the immune protection during infection by regulating host-microbiota (Jukna et al., 2005). We infer that the active ingredient polysaccharide in *C. deserticola* promotes the growth and reproduction of beneficial bacteria in the rumen and reduces the consumption of carbohydrates and other nutrients by pathogenic bacteria, and bring the pH of rumen fluid close to the optimal fermentation range (6.5–7.0) (Nocek, 1997). AS a result, improving the fermentation environment of the rumen and promoting the absorption and utilization of nutrients by the organism, as shown by Spearman correlation and clustering analysis, the higher abundance of *Lactobacillus* corresponded to better BWG and FCR (Figure 5).

Research has found that the active ingredient polysaccharide in alfalfa and shiitake mushroom polysaccharide regulated rumen fermentation in dairy cows and improved the total VFA production (Xue et al., 2015; Li et al., 2018). In the present study, feeding *C. deserticola* significantly increased the ratio of Isobutyrate acid, isovalerate acid, and valerate acid in the rumen fluid, while Spearman correlation and cluster analysis showed positive effect of *Carnobacterium* on the ratio of Isobutyrate acid, isovalerate acid and valerate acid in rumen fluid (Figure 5). *Carnobacterium* is a typical *Lactobacillus* species belonging to the Firmicutes phylum, which is the most abundant genus in animals under heat stress and it produces acid from carbohydrates. Isobutyrate acid and isovalerate acid are commonly referred to as conjugate acids, which promote degradation of food structural carbohydrates (Wilson, 2008) and growth of fiber degrading microbial species, being thus conducive to the digestion of fibers (Liu et al., 2009; Wu et al., 2021). Isobutyrate acid can be used as an energy source by ruminants, and cellulolytic bacteria can use Isobutyrate acid as a substrate to synthesize long-chain fatty acids and aldehydes (Nagaraja, 2016). Studies have shown that Isobutyrate acid supplementation in Simmental cattle increases the concentration of total VFAs in the rumen and improves the digestibility of organic matter, crude protein, and neutral detergent fiber (Liu et al., 2009; Correia et al., 2021). As mentioned earlier, digestibility of DM and OM in the CON group was lower than that in groups that received *C. deserticola* supplementation (Liu et al., 2020), whereas the digestibility of LC and MC groups was significantly higher than that in the CON group (Liu et al., 2020). Therefore, it can be inferred that the addition of *C. deserticola* enhanced the function of *Carnobacterium* in improving feed digestibility. The total VFA concentration in rumen fluid did not change significantly with the increase of *C. deserticola* addition (Liu et al., 2020), probably due to the difference of polysaccharide type and test subjects.

Methane (CH_4_) released by ruminants is the result of the anaerobic fermentation of structural carbohydrates found in forage (Min et al., 2020). The mechanisms affecting the CH_4_ production include the amount of fermentable carbohydrates found in feed and that of VFA produced in the rumen. The amount of acetic acid and propionic acid in the rumen might also affect the production of CH_4_ in ruminants (Xiao, 2016). It has been shown that moderate amounts of shiitake mushroom polysaccharide, Ganoderma lucidum polysaccharide, and Cordyceps sinensis polysaccharide can influence the type of fermentation, increase propionic acid production, thus inhibit the growth of methanogens and significantly reduce the artificial rumen CH_4_ production (Pan, 2019). We did not observe the dominant bacterium *methanobacterium* at the genus level, suggesting that the active ingredient polysaccharide in *C. deserticola* may also have inhibitory effects on the growth of *methanobacteria*. However, the detected CH_4_ emission was increased (Liu et al., 2020). It has also been shown that metabolites of *Lactobacillus* altered the type of ruminal fermentation, leading to a decrease in the proportion of acetic acid in the rumen while increasing that of propionic acid, as well as negatively impacted the supply of H_2_ during CH_4_ synthesis, thus reducing CH_4_ production (Xiao, 2016), and stimulate the growth of ruminal microorganisms, improve nutrient utilization and energy generation (Zhou et al., 2016). Interestingly the study also showed that inhibition of CH_4_ emission was not increased with the increase in *Lactobacillus* abundance in the rumen of grazing sheep, showing an impact only within a certain range (Xiao, 2016). The production of CH_4_ is controlled by a combination of factors. The polysaccharide in *C. deserticola* directly inhibits CH_4_ emission by hindering the growth of methanogenic bacteria and indirectly inhibits CH_4_ emission by increasing the abundance of beneficial bacteria such as Lactobacillus. However, the other factors had a stronger promoting effect than the inhibition by polysaccharides, and the exact reasons need to be further investigated.

It has also been shown that functional polysaccharides have a positive effect on regulating rumen flora structure, Hericium erinaceus polysaccharides can alleviate rumen acidosis and maintain rumen health in goats by regulating rumen flora structure (Li et al., 2019). Fermented wheat bran polysaccharides regulate Dorper × thin-tailed Han crossbred meat lamb’s rumen flora structure and maintain rumen health (Meng, 2018). Artemisia polysaccharide can improve the proportion of rumen Firmicutes and Fibrobacteres and reduce the proportion of pathogenic bacteria (Jin et al., 2021). This could explain the variation in the abundance of Bacteroides as well as members of the genus Firmicutes at the level of the rumen. Bacteroides and Firmicutes are the dominant phyla in the ruminal microbiota (Singh et al., 2012; de Oliveira et al., 2013; Zhou et al., 2017; Bi et al., 2018). Bacteria in the Firmicutes phylum possess a wide variety of metabolic enzymes with activity over a starch, cellulose, hemicellulose, oligosaccharides, and butyric acid (Murphy et al., 2010; Sollinger et al., 2018), thus promoting energy absorption and fat deposition in ruminants The. *C. deserticola* significantly altered the relative abundance of Firmicutes at the genus level for members of groups such as *Lactobacillus*, *Ruminococcaceae_NK4A214_group*, *Christensenellaceae_R-7_group*, etc. The phylum Bacteroidetes plays an important role in the degradation of non-fibrous substances (proteins, polysaccharides, etc.) in the rumen (Pope et al., 2012; Naas et al., 2014), while promoting the development of the host’s immune system as well as maintaining the balance of the intestinal microbiota (Xiao et al., 2020). In this study, high levels of *C. deserticola* resulted in a significant decrease in the relative abundance of Bacteroidetes. Members of Bacteroidetes have higher mean glycoside hydrolases (GHs) and polysaccharide lyases (PLs) genes per genome, as well as signal peptide-containing GHs and PLs (El Kaoutari et al., 2013), compared to the members of the Firmicutes, or any other bacterial phyla in the GI tract, are one of the primary degraders of the many complex polysaccharides in the plant cell wall (Meale et al., 2016). This might explain the significant changes in the relative abundance of Bacteroides in animals fed with *C. deserticola*. The degradation ability of Firmicutes is mainly limited to the cell surface, while degradation by Bacteroides occurs mainly in the periplasm or intracellularly (White et al., 2014). A higher abundance of Bacteroides and Firmicutes can improve the host’s ability to degrade forage resources, thereby improving the host’s adaptability to harsh environments (Han et al., 2020). In the present study, the microbial community in LC group had the highest abundance and uniformity, therefore, it can be deduced that sheep in the LC group had a higher capacity for digesting and metabolizing forage. these findings are consistent with findings of the previous studies that reported the digestibility of DM and OM in sheep of the LC group was significantly higher than that of other experimental groups (Liu et al., 2020). *Christensenellaceae_R-7_group* mainly plays an important role in maintaining immunity and gastrointestinal function of the rumen (He et al., 2019; Liu et al., 2019a,b). The variation in the abundance of *Christensenellaceae_R-7_group* in the text can be explained by the important role played by polysaccharides and phenethyl alcohol glycosides in the immunization of livestock and poultry (Zhao et al., 2017).

In addition, the functional profile of the ruminal microbial community in grazing sheep was predicted by Tax4Fun. The microbial community in the rumen of animals included in the LC group was rich in genes related to flavonoid biosynthesis; therefore, it can be hypothesized that a higher amount of flavonoids is synthesized in the rumen of LC animals. In ruminants, flavonoids are closely associated with lipid metabolism, endocrine, and antioxidant activities, and antibacterial and antiviral effects, thus playing an important role in promoting growth, improving production performance and immunity (Xie and Zhang, 2003; Xie and Zhang, 2005; Yang et al., 2019). In this study, Spearman correlation and clustering analysis showed that the relative abundance of *Ruminococcaceae_NK4A214_group* and *Christensenellaceae_R-7_group* had a positive effect on EE digestibility. *Ruminococcaceae_NK4A214_group* is associated with the degradation of fibrous material and can produce hydrolytic enzymes such as cellulase, which destroys the cell wall of crude fiber (Zeng et al., 2020), thereby improving the utilization of high-fiber diets by ruminants. Moreover, the abundance of *Ruminococcaceae_NK4A214_group* in the LC group was significantly higher compared to the other groups, whereas the relative abundance of *Christensenellaceae_R-7_group* in the MC group was significantly lower than that in other groups. Therefore, in combination with the results of previous studies, the digestibility of EE in the HC group was significantly lower than that in other groups (Liu et al., 2020). In contrast, the digestibility of EE was the highest in the LC group; therefore, it can be deduced that the digestibility of EE in sheep of the LC group was high. However, it is important to acknowledge that metagenomic function prediction analysis might not represent the actual function of rumen microorganisms. Although several rumen-based studies have been conducted in recent years, the complexity of the rumen microbiota has not been completely understood. Therefore, future studies involving transcriptomics, proteomics, and metabolomics should be considered, and more research is required to explore the specific roles of microorganisms found in the rumen.

## Conclusion

Certain levels of *C. deserticola* alter the abundance of rumen bacteria in grazing sheep and affect rumen function, associated with the action of active polysaccharide component. The polysaccharides in *C. deserticola* can improve the rumen fermentation environment, regulate the structure of the bacterial flora, and improve immunity. Therefore, *C. deserticola* (with an optimal rate of 2–4%) can be used as an alternative to veterinary drugs and other chemical drugs to improve the performance of grazing sheep.

## Data Availability Statement

The datasets presented in this study can be found in online repositories. The name of the repository and accession number can be found below: NCBI; PRJNA792216. Submission ID: SUB10855584 and BioProject ID: PRJNA792216, Available online at: https://www.ncbi.nlm.nih.gov/bioproject/PRJNA792216.

## Ethics Statement

The animal study was reviewed and approved by the animal sampling procedure strictly followed the rules and regulations of experimental field management protocols (File Nos: 2010-1 and 2010-2).

## Author Contributions

FH and XZ: conceptualization, methodology, and writing – original draft. FH: data curation, funding acquisition, and supervision. XZ: formal analysis and software. XZ, SC, CZ, WD, XL, and FH: writing, review, and editing. All authors have read and agreed to the published version of the manuscript.

## Conflict of Interest

The authors declare that the research was conducted in the absence of any commercial or financial relationships that could be construed as a potential conflict of interest.

## Publisher’s Note

All claims expressed in this article are solely those of the authors and do not necessarily represent those of their affiliated organizations, or those of the publisher, the editors and the reviewers. Any product that may be evaluated in this article, or claim that may be made by its manufacturer, is not guaranteed or endorsed by the publisher.
